# Simulator Training on Neurointerventional Skill Acquisition in Novices: A Pilot Study

**DOI:** 10.3390/neurolint18010016

**Published:** 2026-01-14

**Authors:** Alexander von Hessling, Tim von Wyl, Dirk Lehnick, Chloé Sieber, Justus E. Roos, Grzegorz M. Karwacki

**Affiliations:** 1 Section of Neuroradiology, Department of Radiology and Nuclear Medicine, Neurocenter, Cantonal Hospital Lucerne, University Teaching and Research Hospital, University of Lucerne, 6000 Lucerne, Switzerland; 2 Faculty of Health Sciences and Medicine, University of Lucerne, 6002 Lucerne, Switzerland; 3 Center for Clinical Research–Biostatistics and Methodology, Lucerne Cantonal Hospital, University of Lucerne, 6002 Lucerne, Switzerland; 4 Department of Radiology and Nuclear Medicine, Luzerner Kantonsspital, University Teaching and Research Hospital, University of Lucerne, 6000 Lucerne, Switzerland

**Keywords:** neurointervention, simulation training, stroke, mechanical thrombectomy, angio-simulator, Mentice, interventional neuroradiology, skill acquisition

## Abstract

Background: Simulation-based training may offer a useful approach to support skill acquisition in neurointerventional stroke treatment without exposing patients to procedural risks. As the global demand for thrombectomy rises, training strategies that ensure procedural competence while addressing workforce constraints are increasingly important. With this pilot study, we aim to generate a hypothesis as to whether additional exposure of trainees to mechanical thrombectomy could benefit from simulator training on top of the standard training carried out on flow models. This study was designed as an exploratory pilot investigation and was not able to provide inferential or confirmatory statistical conclusions. Methods: Six novice participants (advanced clinical-year medical students with completed anatomical and preclinical training, but without previous exposure to catheter-based interventions) performed two neurointerventional tasks, vascular access and mechanical thrombectomy (MTE), on flow models. After a baseline assessment, three participants received standard model-based training (control group), and three received additional simulator training using a high-fidelity angiography simulator (Mentice VIST G5). Performance was reassessed after four weeks using technical and clinical surrogate metrics, which were ranked and descriptively analyzed. Results: No relevant differences were observed between groups for the vascular access task. In contrast, the simulator group demonstrated a trend toward improved performance in the MTE task, with greater gains in efficiency, autonomy, and procedural safety. Conclusions: Our findings indicate a possible benefit of even brief simulator exposure for skill acquisition for complex endovascular procedures such as MTE. While conventional training may suffice for basic skills, simulation may be particularly helpful in supporting learning in more advanced tasks.

## 1. Introduction

Endovascular stroke treatment (EVT) has been established as a safe and effective intervention for acute ischemic stroke, significantly improving functional outcomes when performed in a timely manner [1,2,3]. Over the past decade, the clinical demand for EVT has grown steadily, driven by compelling evidence from large, randomized trials and its integration into international guidelines [4,5,6,7]. As a result, the number of thrombectomies performed worldwide continues to rise [8].

Given the urgent and time-sensitive nature of EVT, healthcare systems are required to ensure 24/7 availability of this treatment in specialized stroke centers. However, this poses significant logistical and personnel challenges. The complexity of EVT demands a high level of technical proficiency, decision-making under pressure, and interdisciplinary coordination. Neurointerventional skills can only be acquired through dedicated training [9]. Simultaneously, legal and institutional regulations mandate sufficient rest periods for medical staff involved in stroke call schedules, further limiting the availability of experienced personnel [10,11].

To address the growing need for adequately trained operators while maintaining compliance with workforce regulations, innovative training approaches must be explored. High-fidelity angiographic simulators have been proposed as one possible method to complement conventional training, which allows for the practice of endovascular procedures in a risk-free and controlled environment and may help trainees develop procedural skills without patient exposure. These simulators replicate realistic anatomical and clinical scenarios, potentially allowing for the practice and refinement of procedural and cognitive skills without patient exposure [12,13,14,15,16,17,18,19].

In this context, we conducted a small pilot study with the intent of generating a hypothesis on whether the use of a modern angio-simulator could potentially facilitate the training of novices in EVT stroke treatment.

Specifically, we aimed to evaluate the potential of simulator-based training to support skill acquisition for key procedural steps in endovascular stroke treatment, i.e., vascular access to the affected vessel and MTE, and its role in complementing traditional hands-on experience.

This study was designed as an exploratory pilot investigation and was not able to allow inferential or confirmatory statistical conclusions.

## 2. Materials and Methods

The Ethics Committee Northwest and Central Switzerland (EKNZ) advised that this project does not fall within the scope of the Human Research Act and, therefore, does not require the approval of the competent ethics committee EKNZ. (BASEC-ID: Req-2025-01706). All participants provided written informed consent prior to inclusion in the study.

General setting and tasks:

Six novice participants were recruited: third-year medical students in a five-year curriculum who had completed coursework in anatomy, physiology, and pathophysiology but had no prior exposure to interventional neuroradiology or catheter-based procedures. They performed two standardized neurointerventional tasks designed for endovascular stroke treatment using two different physical flow models. Students were chosen because they combined foundational anatomical and pathophysiology knowledge with ready availability. Recruitment occurred within the peer group of a student co-author. Consistent with early-stage neurointerventional trainees, such as residents, all participants were inexperienced with catheter-based techniques.

The tasks were designed to simulate two critical steps in the procedure of endovascular stroke treatment:

1. Access task: gaining Access to the brain supplying artery in an original-sized aortic flow model from the femoral to the left internal carotid artery (ICA) using standard technique and materials (model without flow simulation).
2. Mechanical thrombectomy (MTE) task: Removal of a standardized clot from the middle cerebral artery (MCA) in a vascular glass model with simulated blood flow using a balloon catheter, microcatheter, wire, aspiration, and stent retriever.

**Figure 1:**
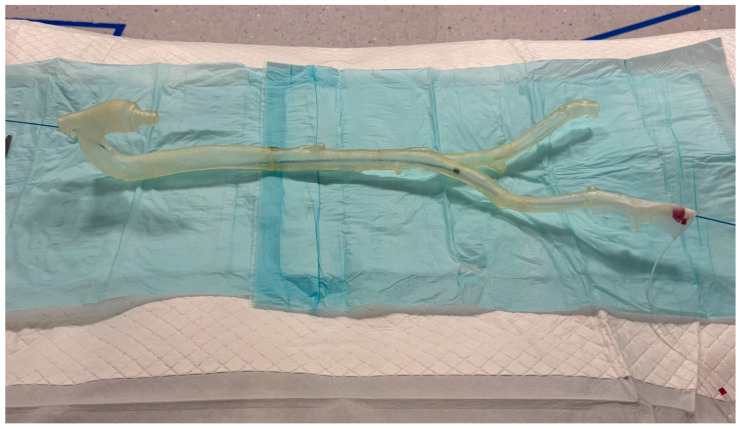
Original-sized 3D-printed resin flow model (type-2 aortic arch) for training vascular access with sheath and 5F-catheter for access to the left ICA.

**Figure 2:**
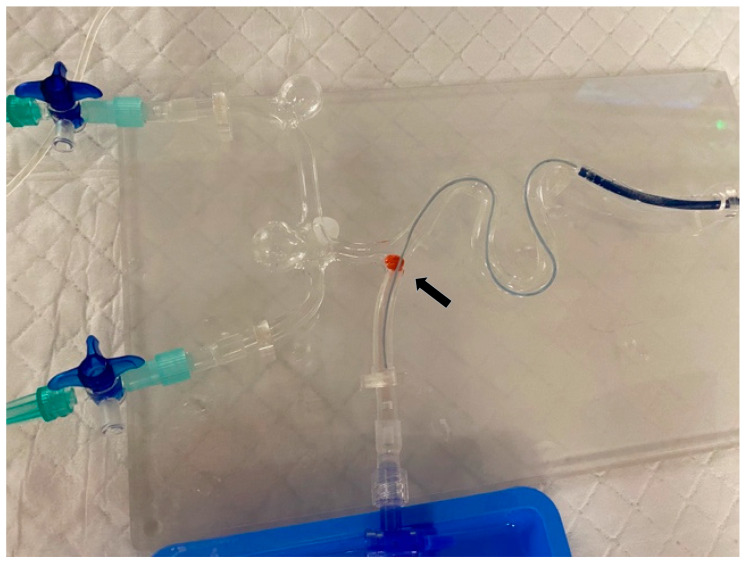
Schematic glass flow model for training of mechanical thrombectomy (standardized, artificial clot [arrow]; 0.021-inch microcatheter is represented in light blue; and balloon guide catheter is represented in dark blue).

Models are depicted in Figure 1 and Figure 2:

Both tasks were conducted under monoplane fluoroscopy with a dedicated biplane neuroangiography suite (Artis Icono, Siemens Healthineers, Forchheim, Germany) and single high-dose exposures when necessary. The experimental setup was arranged in such a way that exposure of the participants to radiation was in no case possible. This was monitored through live radiation exposure surveillance during the experimental setup (with the RaySafe real-time dosimetry system assessing the participants and the space from where the model was operated), and in no single instance was any radiation exposure detected. The Aortic Model is an original-size type II aortic arch model printed on a Laser 3D Printer (Formlabs 1) using transparent resin. The intracranial vascular model is hand-blown from glass tubes with a 4 mm inner diameter, schematic and approximately to scale, modeled after human anatomy, simulating the carotid siphon and major intracranial bifurcation of the ICA. Original neurointerventional materials (5F catheters from Cordis, Terumo, and Traxxess wires, and “Merci” balloon-guide catheter and Solitaire stent retrievers) were used. In the intracranial model, blood flow was simulated using water, which was pumped through the model in the physiological direction by an aquarium pump. Inflating the balloon on the guiding catheter allowed cessation of antegrade flow and, when combined with aspiration, brief flow reversal was obtained. The synthetic clot material was composed of a hydrophilic organic hydrate and calcium sulfate-based materials (Rapid Medical Ltd., Carmel, Yoqneam, Israel) with a standardized length of 5 mm. The first and last authors have over 15 and 10 years of experience, respectively, in catheter-based thrombectomy, along with experience using various flow models and artificial thrombi. The decision to use this artificial thrombus material (in combination with the glass model) was made empirically, compared with other materials such as animal blood, modeling clay, or bell-pepper fragments often used in hands-on workshops, because it replicates the properties of real thrombi in most patients relatively well.

Participant Orientation:

The information of all participants was standardized, identical, and included the following key elements [20]:

Prior to the start of the simulation study, all participants underwent a standardized training session to ensure consistent orientation across the cohort. During this structured briefing, the purpose and objectives of the experiment were explained. Participants were informed about the specific tasks they were expected to perform, the time frame within which these tasks needed to be completed, and the duration allocated for each task segment.

Additionally, participants received detailed information regarding the parameters that would be collected throughout the simulation. This included both technical measurements and performance-related data. The training also covered the use of all available tools (different available catheters and wires), including instructions on how to operate the simulation models and radiographic equipment. Emphasis was placed on understanding how participant decisions could influence radiation exposure (of the flow models), task duration, and the potential outcomes of the simulation. The study supervisor demonstrated both tasks to each participant on the flow models in a standardized way immediately before the baseline measurement. The role of the study supervisors was also clearly defined: they acted solely as observers during the simulation and were instructed to intervene only if participants explicitly requested assistance or if intervention was deemed necessary for safety or procedural reasons. This standardized orientation process aimed to ensure a high degree of consistency and comparability across all participant experiences.

Baseline Assessment

The baseline assessment for both tasks included observing the following measurements:

- Successful completion of the task (yes/no);
- Time needed to complete the task (maximum time for each task was 15 min) measured in minutes;
- Identification and number of errors detected by an experienced neurointerventionalist with 16 years of training (number);
- Number of tips necessary to advance in the task (number) detected by an experienced neurointerventionalist;
- Fluoroscopy time (minutes);
- Dose area product (DAP, Gy·cm^2^).

In the second task, the following measurements were additionally observed:

- Number of attempts to successful MTE (or expiration of the given time frame);
- Number of embolizations into new territories (ENT);
- Final thrombolysis in cerebral infarction (TICI) score.

Grouping and rank-based assessment

After the baseline assessment, participants were split into two matched groups: a **control** group (*n* = 3) trained only on the two physical models used with a training time of 90 min each, and a **simulator** group (*n* = 3) trained on both physical models (with a training time of 90 min) as well as the Mentice angio-simulator for another 90 min.

Participants were assigned to either the simulator group or the control group with the goal of creating two groups of comparable baseline performance. To achieve this, group allocation was based on the outcome parameters: task success and time required to complete the tasks. All participants successfully completed the vascular access task, whereas two participants failed to complete the MTE task. These two were deliberately assigned to different groups to ensure balance in baseline success. The remaining four participants were ranked according to their baseline task completion time, and then alternately assigned to either group to further balance group performance.

The decision to use rank-based evaluations rather than changes in arithmetic means was methodologically motivated. Changes in means or sums can be misleading, especially in small samples, and are mathematically unstable when individual trajectories diverge.

The Simulator

**Figure 3:**
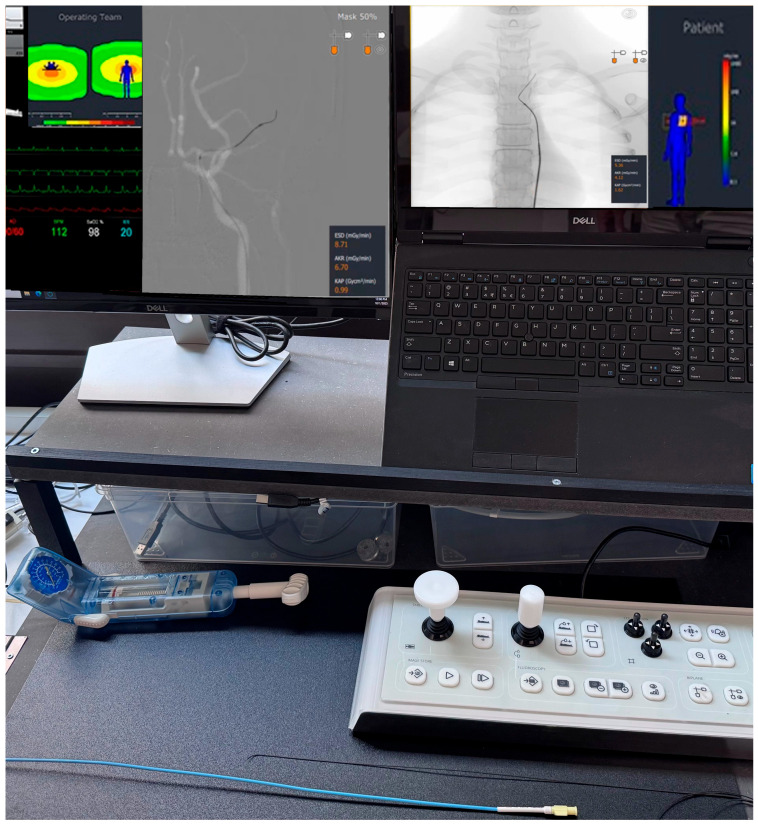
Mentice Vist G5 angio simulator (www.mentice.com).

The Mentice VIST G5 (Figure 3) is an image-guided endovascular VR simulator designed for hands-on procedural training for medical professionals across neurovascular, interventional radiology, and interventional cardiology domains. The platform provides haptic-enabled catheter and wire manipulation with realistic device–vessel interaction, while emulating angiographic imaging and workflow to support task rehearsal and decision-making in a radiation-free environment. A broad library of procedure modules, including acute ischemic stroke thrombectomy interventions, is available, enabling graduated training scenarios that target technical and cognitive skills. For our training purpose, we used the neurovascular “Stroke” module with different stroke scenarios of the anterior circulation (https://www.mentice.com/software/nv-acute-ischemic-stroke-ais-cases; accessed on 8 January 2026). During the simulation, the simulator provides feedback on errors made (e.g., when catheters are pushed without wire support, or when thrombectomy was performed without aspiration) and usage of simulated radiation and fluoroscopy times.

Flow model and simulator training

Following the introduction and baseline assessment, all six participants were given a four-week period in which they had 90 min available to independently repeat and practice the two tasks from the baseline measurement on the two physical flow models. Technical but no procedural support was given during this period.

The participants who were assigned to the simulator intervention group (*n* = 3) received an additional 90 min training opportunity using the endovascular simulation platform. Prior to this simulator training, a standardized orientation was provided to these three participants, covering the functionality and operation of the simulator. Participants received a 15 min demonstration of a virtual stroke thrombectomy conducted by the study supervisor on the simulator. This demonstration included simulated endovascular access to the left common carotid artery, followed by a stroke simulation showcasing a successful MTE procedure targeting an occlusion in the MCA. Subsequently, the three participants in the simulator group were given 90 min of self-directed hands-on training using a simulated MCA occlusion scenario, during which they could practice both arterial access and thrombectomy technique.

Although participants in the simulator group had a longer overall training time—which precludes a precise separation of the qualitative benefit of simulator training from the effect of increased training time per se—in everyday practice, the time at the flow model cannot readily be expanded because access to an angiography suite is limited. Instead, it is more feasible to add simulator sessions in addition to flow-model training. Accordingly, our chosen methodology more closely reflects real-world conditions and measures the effect of simulator training as an “on-top” adjunct to standard flow-model training.

Final Assessment

Four weeks after the baseline assessment, all six participants repeated both tasks under identical conditions on the two flow models. The same performance metrics as in the baseline assessment were recorded.

All tasks were recorded on video, ensuring that participants were not identifiable (only hands and forearms were visible). The evaluation of task performance was conducted from the video recordings, and the assessor was blinded to group allocation.

**Figure 4:**
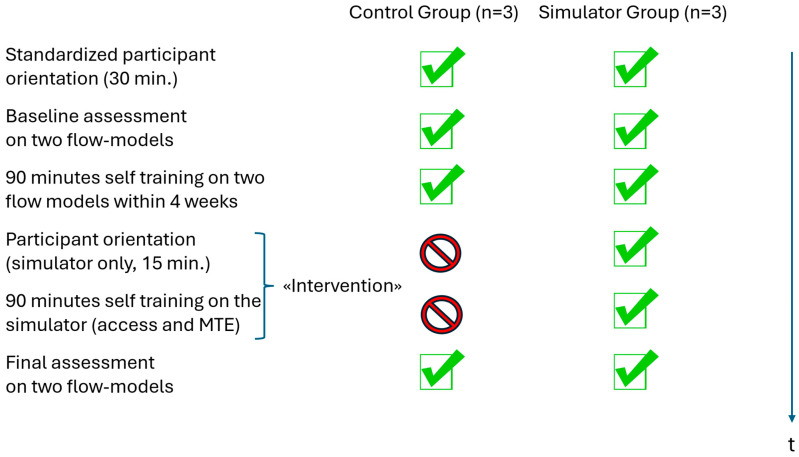
Flow chart of the pilot study. This sequence was deliberately chosen (to reflect our everyday training situation) with the use of the simulator serving as the “intervention” that differentiates the two groups.

The entire timeline of the pilot study and tasks fulfilled by both groups is shown in Figure 4.

## 3. Statistical Analysis

To enable comparison despite the small sample size and heterogeneous performance metrics, each task parameter was summarized using a rank-based system. For each parameter, participants were ranked from one (best performance) to six (worst performance). In cases where two or more participants achieved identical values, the average of the respective ranks was assigned. Rank sums were calculated separately for each task, and time point to allow descriptive comparison between the simulator and control groups. No formal hypothesis testing was performed due to the exploratory nature of the study.

All measured parameters were equally weighted.

## 4. Results

Table 1 shows the results of the baseline and final assessment of both groups and tasks.

Table 2 shows the conversion of the measured values (from Table 1) into ranks. For example, among the six participants included, from the baseline measurement, participant number one took the fifth-longest time to complete the access task (3.73 min) and, therefore, received rank five in this category (represented by the red star in Table 2 below). As another example, in the MTE task, participant number 5 from the simulator group had the shortest fluoroscopy time in the final assessment (2.8 min) and, therefore, achieved rank one in this category (represented by the yellow star in Table 2 below).

**Figure 5:**
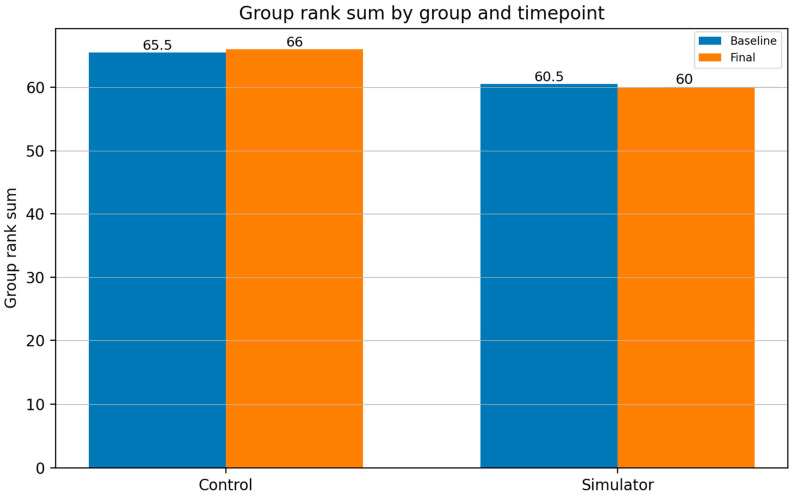
Summarized ranks of both groups and timepoints for Task 1 “Access”. The rank sums in both groups are similar at baseline and at the final assessment after the training period. No advantages of the simulator group can be identified.

Figure 5 shows the rank sums of both groups for Task 1 “Access” at baseline and final measurement.

**Figure 6:**
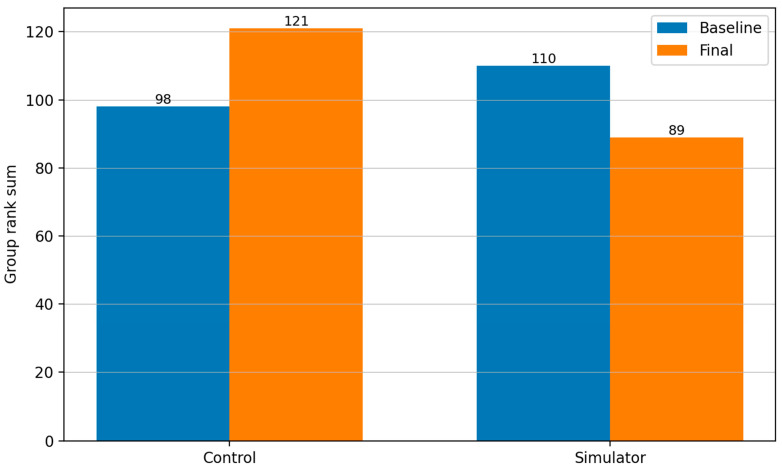
Summarized ranks of both groups and timepoints for Task 2 “MTE”. The simulator group appeared to exhibit a trend of greater training progression and rank shift in favor of the simulator group.

Figure 6 shows the rank sums of both groups for Task 2 “MTE” at baseline and final measurement.

For Task 1 (access), no differences in training progression were observed between participants in the simulator group and those in the control group. The rank sums of both groups were similar at baseline (simulator group: 60.5 vs. control group: 65.5) and remained nearly unchanged at the final visit after the training period (simulator group: 60 vs. control group: 66).

For Task 2 (MTE), the simulator group appeared to exhibit a trend toward greater training progression based on descriptive rank analysis compared to the control group. At baseline, participants in the control group initially showed a slight performance advantage (simulator group: 110 vs. control group: 98). However, this was reversed by the final visit: participants in the simulator group showed lower rank sums (simulator group: 89 vs. control group: 121), suggesting a relative performance advantage in this sample, though this is not statistically confirmed.

This improvement was reflected consistently across most evaluation parameters for Task 2 and was evident in most individual metrics. No difference was observed for embolizations in new territories (ENT), while only minor shifts in favor of the simulator group were seen in MTE attempts and successes. In contrast, more pronounced improvements favoring the simulator group were found for these parameters: time −2.5 (rank sum), tips −5 (rank sum), and errors −4 (rank sum). The control group improved slightly, but not to the same extent. This is reflected by the change in the rank sums: time +2.5, tips +5, errors +4.

**Figure 7:**
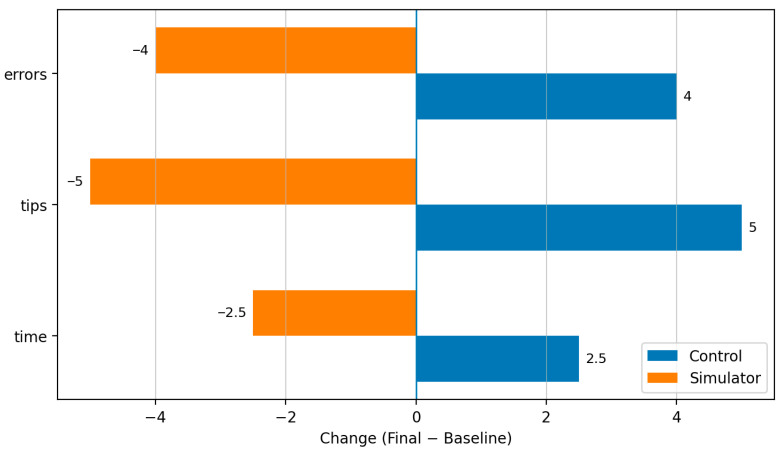
Change in ranks between the two groups from baseline to final assessment with respect to the parameters errors, tips, and time.

Figure 7 shows the change in ranks between both groups from baseline to final assessment.

## 5. Discussion

This pilot study investigated the effect of high-fidelity simulation training on the performance of novices (third-year medical students) in endovascular stroke treatment, with a focus on two procedural tasks: vascular access (Task 1) and MTE (Task 2). The evaluation was based on rank sum comparisons to account for the small sample size and the heterogeneous nature of the performance metrics. When analyzing both tasks separately, no relevant differences in training progression were observed for the access task. Rank sums at baseline and final assessment were nearly identical in both groups, indicating that while participants may have improved individually, the relative group performance remained unchanged. This suggests that conventional training using physical models may be sufficient for acquiring fundamental access skills in this context. In contrast, the MTE task revealed notable advantages for the simulator group. While the control group showed a slightly better performance at baseline, this pattern was reversed at the final visit. Most evaluation parameters showed descriptively favorable changes in the simulator group, including procedure time, number of errors, and external guidance requests. These patterns may point to the potential benefit of simulation training on more complex, technically demanding neurointerventional tasks, though confirmation in larger studies is required.

The use of ranks allowed for a fair and comparable aggregation of heterogeneous parameters, regardless of their underlying scale, while avoiding overemphasis on any single metric such as total time. As noted, the combined time across both tasks would be dominated by MTE complexity and number of attempts, which can distort interpretation. Furthermore, by assigning equal weight to all parameters in the rank sum analysis, we followed a neutral aggregation strategy. While future studies may consider weighted schemes based on clinical relevance, such decisions introduce potential bias. For exploratory studies like ours, equal weighting provides a transparent and reproducible approach.

The strength of our pilot study includes the following points:

- Methodological rigor in a small sample setting: The study employed a careful matching strategy based on baseline performance and used rank-based metrics, which are more robust in small-sample analyses with heterogeneous data.
- Task-specific analysis: The decision to analyze Task 1 and Task 2 separately allowed for a nuanced understanding of how simulation impacts different procedural skill levels.
- Transparent aggregation strategy: Equal weighting of evaluation parameters avoided arbitrary bias and enabled fair comparison of performance.
- Empirical challenge to expectations: The absence of simulator-related benefits in the simpler access task—despite initial assumptions—demonstrates the importance of data-driven conclusions over intuitive expectations.

In contrast, we observed the following limitations:

- Small Sample Size: The limited number of participants (*n* = 6) restricts statistical power and precludes confirmatory conclusions.
- Lack of Blinding: Full blinding of observers and participants was not possible, introducing the potential for observer bias.
- Short Follow-Up: The study measured only short-term training effects, with no data on skill retention or transfer to clinical practice.
- Use of Surrogate Metrics: Performance was assessed through proxy measures (e.g., time, error counts, guidance requests), rather than clinical outcomes.
- Generalizability: As a pilot study with novice participants (third-year medical students), the findings may not generalize to more experienced learners or real-world procedural contexts.

Our findings should be considered hypothesis-generating rather than confirmatory. Nevertheless, the observable performance patterns, particularly in the complex MTE task, warrant further investigation.

## 6. Conclusions

This pilot study allows us to hypothesize that even limited exposure to high-fidelity angiographic simulation could contribute meaningfully to skill acquisition in neurointerventional training in novices (represented as third-year medical students), particularly for complex, cognitively demanding tasks such as mechanical thrombectomy. While no significant advantage was observed for basic vascular access skills, the simulator group showed patterns suggesting increased efficiency, autonomy, and procedural safety during advanced tasks compared to standard training alone. These preliminary findings could inform future studies assessing the role of simulation-based training in early neurointerventional curricula and underscore the need for validation of these preliminary results and optimization of the instructional design of simulator training. Additionally, our study offers a structured model for performance comparison in small-sample settings.

## Figures and Tables

**Table 1:** Results of control and simulator groups for both tasks at baseline and final assessments.

Participant Number	1	3	6	2	4	5	1	3	6	2	4	5	Metrics
**Group**	Control	Control	Control	Simulator	Simulator	Simulator	Control	Control	Control	Simulator	Simulator	Simulator	
**Task 1: Access**	Baseline	Baseline	Baseline	Baseline	Baseline	Baseline	Final	Final	Final	Final	Final	Final	
Time	3.73	2.22	2.83	1.63	6.73	2.03	8.87	2.25	2.38	4.80	1.37	1.28	Minutes
Tipps	3	2	0	0	4	0	3	0	1	1	1	0	Number
Errors	0	0	0	0	0	0	0	0	0	0	0	1	Number
Success	Yes	Yes	Yes	Yes	Yes	Yes	Yes	Yes	Yes	Yes	Yes	Yes	Yes/No
DAP	0.01	0.006	0.01	0	0.06	0.01	0.02	0.01	0.01	0.03	0.01	0.01	Gy·cm^2^
Fluoro-Time	3.2	2.2	2	1.6	6.5	2	3.7	2.2	2.2	3.9	1.2	1.25	Minutes
**Task 2: MTE**	Baseline	Baseline	Baseline	Baseline	Baseline	Baseline	Final	Final	Final	Final	Final	Final	
Time	11.22	15.00	6.55	13.80	15.00	5.87	6.12	15.00	6.12	5.17	12.63	4.00	Minutes
Catheter Changes	0	0	0	0	0	0	0	1	0	0	0	0	Number
Tipps	7	13	4	9	15	5	5	10	3	1	3	3	Number
Errors	4	1	0	2	1	2	1	2	0	0	0	0	Number
MTE attempts	2	3	1	2	2	1	1	2	1	1	3	1	Number
ENT	0	0	0	1	0	0	0	0	0	0	1	0	Number
TICI-Score	3	0	3	2c	0	3	3	0	3	3	3	3	
Success	Yes	No	Yes	Yes	No	Yes	Yes	No	Yes	Yes	Yes	Yes	Yes/No
DAP	0	0.05	0.04	0.03	0.06	0.02	0.02	0.04	0.06	0.01	0.04	0.02	Gy·cm^2^
Fluoro-Time	7.2	8.1	6	8.5	10.4	5.5	4.84	8.7	4.71667	3.8	8.7	2.8	Minutes

The green background highlights the results of the control group; the blue background highlights the results of the simulator group. The orange background highlights the measured parameters of Task 1, and the pink background highlights the measured parameters of Task 2.

**Table 2:** The measured values were converted into ranks for both tasks, groups and timepoints.

Participant Number	1	3	6	2	4	5	1	3	6	2	4	5
**Group**	Control	Control	Control	Simulator	Simulator	Simulator	Control	Control	Control	Simulator	Simulator	Simulator
**Task 1: Access**	Baseline	Baseline	Baseline	Baseline	Baseline	Baseline	Final	Final	Final	Final	Final	Final
Time	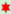 5.0	3.0	4.0	1.0	6.0	2.0	6.0	3.0	4.0	5.0	2.0	1.0
Tipps	5.0	4.0	2.0	2.0	6.0	2.0	6.0	1.5	4.0	4.0	4.0	1.5
Errors	3.5	3.5	3.5	3.5	3.5	3.5	3.0	3.0	3.0	3.0	3.0	6.0
Success	3.5	3.5	3.5	3.5	3.5	3.5	3.5	3.5	3.5	3.5	3.5	3.5
DAP	4.0	2.0	4.0	1.0	6.0	4.0	5.0	2.5	2.5	6.0	2.5	2.5
Fluoro-Time	5.0	4.0	2.5	1.0	6.0	2.5	5.0	3.5	3.5	6.0	1.0	2.0
**Task 2: MTE**	Baseline	Baseline	Baseline	Baseline	Baseline	Baseline	Final	Final	Final	Final	Final	Final
Time	3.0	5.5	2.0	4.0	5.5	1.0	3.5	6.0	3.5	2.0	5.0	1.0
Catheter Changes	3.5	3.5	3.5	3.5	3.5	3.5	3.0	6.0	3.0	3.0	3.0	3.0
Tipps	3.0	5.0	1.0	4.0	6.0	2.0	5.0	6.0	3.0	1.0	3.0	3.0
Errors	6.0	2.5	1.0	4.5	2.5	4.5	5.0	6.0	2.5	2.5	2.5	2.5
MTE attempts	4.0	6.0	1.5	4.0	4.0	1.5	2.5	5.0	2.5	2.5	6.0	2.5
ENT	3.0	3.0	3.0	6.0	3.0	3.0	3.0	3.0	3.0	3.0	6.0	3.0
TICI-Score	2.0	4.5	2.0	4.0	4.5	2.0	3.0	6.0	3.0	3.0	3.0	3.0
Success	2.5	5.5	2.5	2.5	5.5	2.5	3.0	6.0	3.0	3.0	3.0	3.0
DAP	1.0	5.0	4.0	3.0	6.0	2.0	2.5	4.5	6.0	1.0	4.5	2.5
Fluoro-Time	3.0	4.0	2.0	5.0	6.0	1.0	4.0	5.5	3.0	2.0	5.5	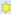 1.0

(The red and yellow stars refer to the examples mentioned in the text). The green background highlights the results of the control group; the blue background highlights the results of the simulator group. The orange background highlights the measured parameters of Task 1, and the pink background highlights the measured parameters of Task 2. The bold values represent the summarized rank values of individuals, and the group rank sums below the parenthesis.

## Data Availability

The original contributions presented in this study are included in the article. Further inquiries can be directed to the corresponding author.
